# Colib’read on galaxy: a tools suite dedicated to biological information extraction from raw NGS reads

**DOI:** 10.1186/s13742-015-0105-2

**Published:** 2016-02-11

**Authors:** Yvan Le Bras, Olivier Collin, Cyril Monjeaud, Vincent Lacroix, Éric Rivals, Claire Lemaitre, Vincent Miele, Gustavo Sacomoto, Camille Marchet, Bastien Cazaux, Amal Zine El Aabidine, Leena Salmela, Susete Alves-Carvalho, Alexan Andrieux, Raluca Uricaru, Pierre Peterlongo

**Affiliations:** GenOuest Core Facility, UMR6074 IRISA CNRS/INRIA/Université de Rennes 1, Campus de Beaulieu, 35042 Rennes Cedex France; BAMBOO team, INRIA Grenoble Rhône-Alpes & Laboratoire Biométrie et Biologie Évolutive, UMR5558 CNRS, Université Claude Bernard (Lyon 1), Campus de la Doua, 43 Boulevard du 11 Novembre 1918, Villeurbanne Cedex, 69622 France; MAB team, UMR5506 CNRS, Université Montpellier II, Sciences et techniques, Université Montpellier 2 LIRMM UMR 5506 CC477 161 rue Ada, Montpellier, 34095 Cedex 5 France; INRIA/IRISA, Genscale team, UMR6074 IRISA CNRS/INRIA/Université de Rennes 1, Campus de Beaulieu, Rennes, 35042 Cedex France; Department of Computer Science and Helsinki Institute for Information Technology HIIT, University of Helsinki, Helsinki, FI-00014 Finland; University of Bordeaux, LaBRI/CNRS, Talence, F-33405 France; University of Bordeaux, CBiB, Bordeaux, F-33000 France

**Keywords:** NGS, Metagenomics, RNA-seq, Variant calling, Whole-genome assembly-less treatment, De Bruijn graph, Bloom filter, long read correction

## Abstract

**Background:**

With next-generation sequencing (NGS) technologies, the life sciences face a deluge of raw data. Classical analysis processes for such data often begin with an assembly step, needing large amounts of computing resources, and potentially removing or modifying parts of the biological information contained in the data. Our approach proposes to focus directly on biological questions, by considering raw unassembled NGS data, through a suite of six command-line tools.

**Findings:**

Dedicated to ‘whole-genome assembly-free’ treatments, the Colib’read tools suite uses optimized algorithms for various analyses of NGS datasets, such as variant calling or read set comparisons. Based on the use of a de Bruijn graph and bloom filter, such analyses can be performed in a few hours, using small amounts of memory. Applications using real data demonstrate the good accuracy of these tools compared to classical approaches. To facilitate data analysis and tools dissemination, we developed Galaxy tools and tool shed repositories.

**Conclusions:**

With the Colib’read Galaxy tools suite, we enable a broad range of life scientists to analyze raw NGS data. More importantly, our approach allows the maximum biological information to be retained in the data, and uses a very low memory footprint.

**Electronic supplementary material:**

The online version of this article (doi:10.1186/s13742-015-0105-2) contains supplementary material, which is available to authorized users.

## Findings

### Background

For some years now, owing to the impact of high-throughput sequencing, also known as next-generation sequencing (NGS), genomics is witnessing profound changes. NGS technologies generate huge volumes of data, up to terabyte scale, and new types of raw and processed data. Usually, a generic assembly (preprocessing) is first applied to the raw sequences, and then, in a second step, the information of interest is extracted. This protocol may lead to a significant loss of information, or may generate chimerical results because of the heuristics used in the assembly. To circumvent this problem, we developed a set of innovative methods for extracting information of biological interest directly from NGS data, which allows the user to bypass a costly and often inaccurate assembly phase. Notably, the approaches developed do not require the availability of a reference genome. This considerably broadens the spectrum of applications. In this paper we present our approach, which relies on a tools suite born from the Colib’read [1] project and is dedicated to whole-genome assembly-free treatments. A set of six tools based on this framework, KISSPLICE [2], MAPSEMBLER2 [3], DISCOSNP [4], TAKEABREAK [5], COMMET [6], and LORDEC [7], are described below. To facilitate the use and the dissemination of our approach, we have developed Galaxy [8–11] tools and created repositories on GUGGO Tool Shed [12, 13]. We first highlight the fundamental computational concepts shared by the tools, and this is followed by the algorithmic developments and tool descriptions. Next, several applications using biological data are described and the Galaxy integration and dissemination processes are then detailed. Finally, the Galaxy integration and processes are described.

### Overview

**Fig. 1:**
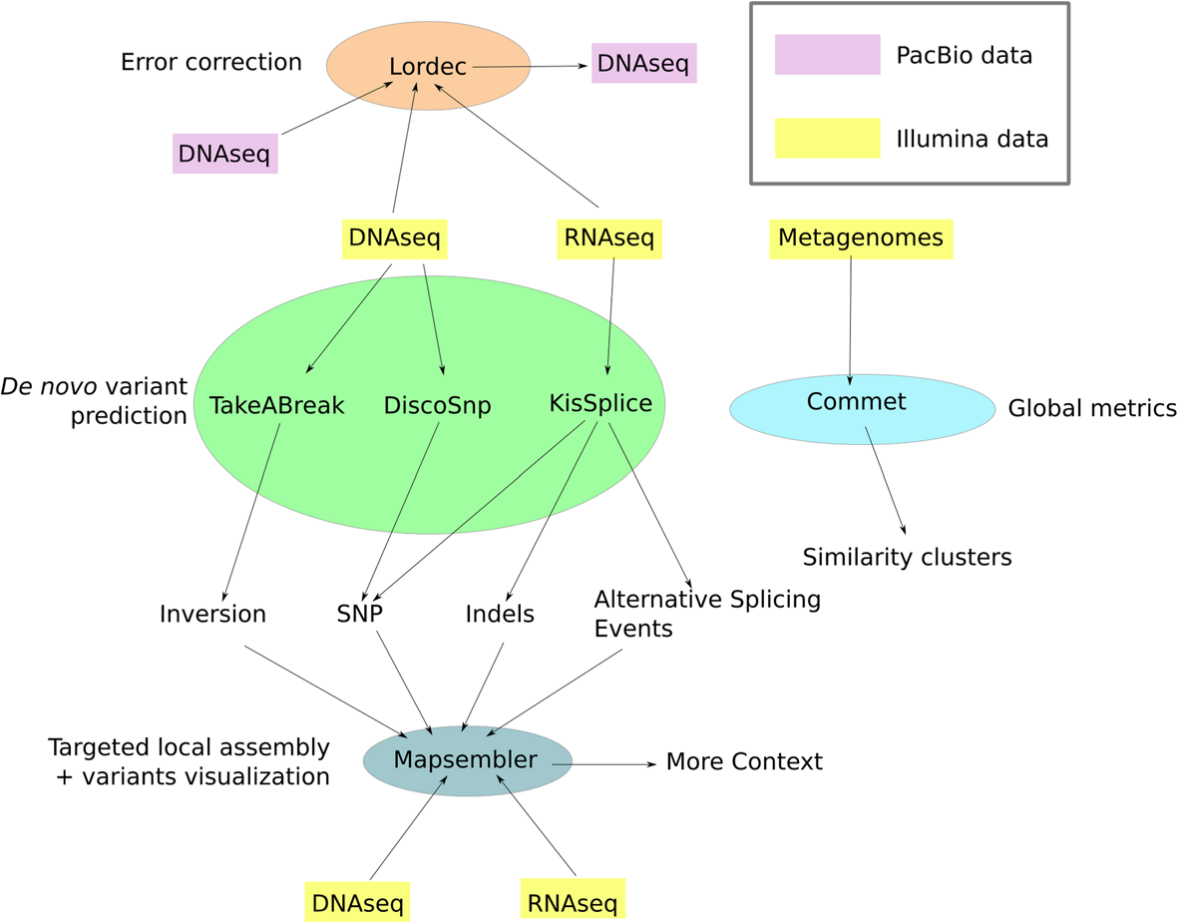
Overview of the six tools from the Colib’read project integrated with Galaxy and presented in this paper

The common denominator of all the tools presented is the fact that they are all dedicated to the analysis of NGS datasets without the need for any reference genome. An overview of these six tools is presented graphically in Fig. 1. Table 1 summarizes the inputs and outputs of each tool. In short, KISSPLICE, DISCOSNP, and TAKEABREAK perform *de novo* variant identification and quantification. For these tools the general approach is: 1) define a model for the elements sought; 2) detect in one or several NGS datasets those elements that fit the model; 3) output these together with a score and their genomic neighborhood. MAPSEMBLER2 generates a targeted assembly surrounding sequences of interest provided by the user. MAPSEMBLER2 can provide the assembly as a graph and proposes a tool for visualizing it.

**Table 1:** Summary of the Colib’read tools inputs and outputs

Tool	In	Out
KISSPLICE	One or more RNA-seq read set(s)	SNPs, small indels, alternative splicing events
DISCOSNP	One or more raw genomic read set(s)	SNP sequences with their coverages
TAKEABREAK	One or more raw genomic read set(s)	Inversion breakpoints
MAPSEMBLER2	Pieces of known sequences, and associated raw read sets	Validation and visualization of genome structure near a locus of interest
COMMET	Several raw metagenomic complex read sets	Global comparison of input sets at the read level
LORDEC	Illumina and PacBio read sets	Corrected PacBio read set

LORDEC uses short reads for correcting third-generation long reads, and finally COMMET (COmpare Multiple METagenomes) is dedicated to the comparison of numerous metagenomic read sets. Special care was given to limit both the memory and time requirements of all tools. Thus, five of the six tools are based on the usage of a compact representation of a de Bruijn graph, as explained in the next section.

Note that all the algorithms presented here were developed bearing in mind the need for simple and user-friendly tools. They can be applied on raw sequenced short reads, without requiring any pretreatment. However, if users are aware of bias such as contaminants or systematic sequencing errors, they can be used on preprocessed datasets and this can give better results.

### A common kernel: the de Bruijn graph

**Fig. 2:**
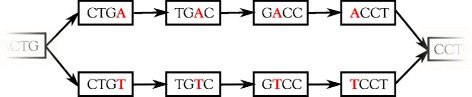
Toy example of a ‘bubble’ in the de Bruijn graph (*k*=4). The bubble is generated by an SNP present in two polymorphic sequences, …CTGACCT… and …CTGTCCT…

**Fig. 3:**
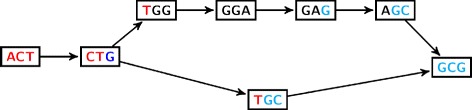
de Bruijn graph with *k*=3 for the sequences:  (*awb*) and  (*ab*). The pattern in the sequence generates an (*s,t*)-bubble, from  to . In this case, *b*=  and *w*= GGA have their first letter  in common, so the path corresponding to the junction *ab* has *k*−1−1=1 vertex

From a computational viewpoint, with the exception of COMMET (which has no need of such a graph, and only uses a bloom filter), all the algorithms presented are based on the use of a de Bruijn graph (dBG). A dBG is a directed graph whose vertices are the *k*-long words contained in the reads, i.e. *k*-mers, and whose arcs represent all *k*-1 overlaps between these *k*-mers (vertices). See Figs. 2 and 3 for examples of dBGs.

Through the last decade, dBGs have been used extensively in the short read assembly framework. Indeed, the construction of such graphs is fast as it avoids any alignment computation, and it is memory efficient, as it compresses the read redundancy. In addition, since every nucleotide is explicitly present in this structure, sequence variants correspond to recognizable patterns. Therefore dBGs are well tailored for developing methods for detecting sequence polymorphism. DISCOSNP detects patterns generated by single-nucleotide polymorphisms (SNPs); KISSPLICE deals with RNA-seq data and finds patterns generated by SNPs, indels, and alternative splicing (AS) events; and TAKEABREAK detects patterns generated by inversions.

MAPSEMBLER2 and LORDEC are also based on a dBG, respectively for building a targeted assembly and as a reference for correcting third-generation long reads.

With the exception of KISSPLICE and COMMET, all the algorithms presented are based on an efficient representation of the de Bruijn graph with optimized bloom filters, implemented in the GATB C++ library [14], as used for the first time in the MINIA [15, 16] assembler.

A bloom filter is a probabilistic data structure that stores the presence/absence of items. It consists of a simple bit vector, initially all set to ‘0’. Any item is associated with a set of a few addresses in this vector (typically seven addresses). While adding an item, the corresponding bits are set to ‘1’. Note that a bit may be set to ‘1’ from several distinct items. While querying an item, if all its bits are equal to ‘1’ then the item is considered as indexed in the bloom filter. Conversely, if any of its bits are equal to ‘0’, the item is absent from the indexed data. The main advantage of the bloom filter is its simplicity and its low memory footprint. Its main disadvantage is that it is probabilistic: if the bloom filter answers ‘yes’ while querying the presence of an item, this answer may be wrong (with a controlled percentage).

Thus, the bloom filter representation has the main advantage of a very low memory footprint. For instance, nearly 3 billion reads (100 bp) were analyzed by DISCOSNP, using at most 5.7 GB of memory. Moreover, the low memory footprint does not imply a degradation in the running time. The COMMET tool, being a heuristic based only on a bloom filter, is also fast and has an extremely low memory footprint. Unfortunately the GATB [14] data structure does not yet allow the assignment of additional information to dBG nodes. In the KISSPLICE case, as presented in more detail in the following section, the nodes of the graph need to be tagged, thus requiring the use of an explicit dBG representation. Even if this representation is more resource intensive, it scales up perfectly in RNA-seq data problems.

### Description of tools

#### DISCOSNP

DISCOSNP [4] is a reference-free SNP calling program that focuses on the detection of both heterozygous and homozygous isolated SNPs, from any number of sequencing datasets.

The DISCOSNP method rests on the following observation: in the dBG, a SNP generates a pair of paths composed of *k* vertices, which represent 2*k*-1 length sequences that are polymorphic on one position. This corresponds to a so-called bubble in the dBG, as depicted in Fig. 2. The model formalism can be found in [4].

DISCOSNP is composed of two modules: KISSNP2, followed by KISSREADS. The tool takes as input any number (potentially one) of read sets, i.e. samples. It processes all read sets together (creating the dBG and detecting the SNP-specific motifs) and outputs all isolated SNPs (for a given *k*) shared by any number of samples. The KISSNP2 output is a multi-FASTA file in which every consecutive pair of sequences corresponds to the two paths of a SNP (2*k*-1 sequences) together with their left and right contigs, which are reconstructed with the MINIA assembler [15]. The KISSREADS module maps back input reads on the sequences of the predicted SNPs in order to validate them and to provide per allele coverage and per read set information. The coverage is then used to compute a phi score, i.e. a normalized chi-squared statistic varying between 0 and 1, which ranks best those SNPs that are discriminant between the samples. Finally, SNPs are sorted according to the phi score.

DISCOSNP outperforms, mostly in terms of time and memory resources, state-of-the-art *de novo* or reference-based SNP discovery methods [4]. Indeed, DISCOSNP scales remarkably well on big data studies as illustrated in Table 2.

**Table 2:** Time and memory consumption examples

Tool	Sample type	Number of reads	Computation time	Max. RAM use
KISSPLICE	*H. sapiens* tissues RNA-seq	71 million	3 h	8 GB
DISCOSNP	*S. cerevisiae* WGS	1.4 billion	34 h	6 GB
MAPSEMBLER2	*S. cerevisiae* WGS	430 million	24 h	1 GB
TAKEABREAK	*S. cerevisiae* WGS	430 million	2 h	4 GB
COMMET	Soil and seawater metagenomes	71 million	14 h	7 GB
LORDEC	*E. coli* WGS	11 million and 0.08 million	3.3 h	0.66 GB
LORDEC	*S. cerevisiae* WGS	2.25 million and 0.26 million	25 h	0.74 GB

#### KISSPLICE

**Fig. 4:**
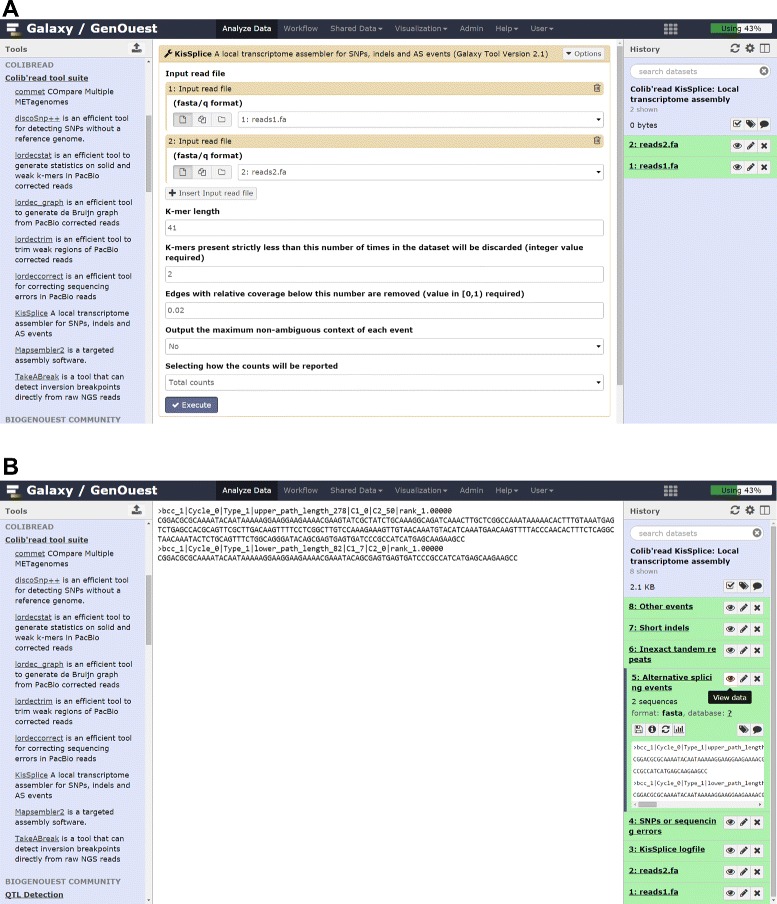
Running KisSplice on Galaxy. **a** KisSplice tool form allowing selection of input datasets and tool parameters. **b** KisSplice outputs

KISSPLICE [2] (Fig. 4) is a program that enables the analysis of RNA-seq data with or without a reference genome or transcriptome. It is an exact local transcriptome assembler that allows identification of SNPs, indels, and AS events. The software can deal with an arbitrary number of biological factors, and it is able to quantify each variant in each condition. KISSPLICE has been tested on Illumina datasets of up to 1 billion reads. The memory consumption is around 5 Gb for 100 million reads. The local aspect of KISSPLICE allows it to scale better to larger datasets than traditional global assemblers, for example Trinity [17]. However, it does not reconstruct full-length transcripts, but only the variable regions. For instance, in an exon skipping event only the sequence of the skipped exon (plus some fixed-length context) is computed.

Variations in a transcriptome (including AS events) correspond to recognizable patterns in the dBG [2], known as ‘bubbles’ as briefly described in the DISCOSNP section above.

An example of such a bubble is given in Fig. 3.

The KISSPLICE program is composed of four steps: (i) de Bruijn graph construction, (ii) biconnected components decomposition, (iii) bubble enumeration, and (iv) event classification and quantification. In the first step, common to other Colib’read tools, the dBG is built from the set of reads using the GATB structure.

The second step in KISSPLICE decomposes the dBG into biconnected components (see [2] for formal definitions). This step requires marking the nodes and cannot be performed with the current version of the GATB structure, explaining why an explicit representation of the dBG is required. This decomposition has the advantage of not splitting the searched motifs while offering the possibility of performing the motif search in each component independently, and possibly in parallel. With RNA-seq data, this lossless graph decomposition is very efficient for splitting the dBG. For DNA-seq data, this decomposition is not efficient as most of the graph is made of a single biconnected component.

The third step, bubble enumeration, is the core of the KISSPLICE program. In this step the goal is to find all motifs (bubbles) satisfying the model constraints. This step is implemented using the enumeration algorithm given in [18].

Finally, in the fourth step each bubble is classified into four categories (indels, SNPs, AS events, and repeats) and quantified in each condition, independently. The quantification is done with KISSREADS, where we obtain the number of reads for each condition mapping to each variant. The final result, i.e. for each event the sequence of the variable part plus some sequence context and the quantification, is then output in a FASTA format.

#### TAKEABREAK

TAKEABREAK [5] is a method of detecting inversion variants from one or several sets of reads without any reference genome. The rationale behind it is similar to that of DISCOSNP: inversion variants generate particular topological motifs in the dBG.

Inversion variants are defined as follows: a sequence *I* is said to be an inversion variant between two genomes if we can find the sequence *aIb* in one genome and *aI*’*b* in the other, with *a* and *b* being two k-mers and *I*’ being the reverse complement of *I*. We define the k-mers *u* and *v* as the first and last k-mers of *I* respectively. The occurrence in the data of the four breakpoint sequences *au*, *vb*, *av*’, and *u*’*b* (each of size 2*k*) generate a particular motif in the dBG that we call the inversion pattern. This motif is composed of two *k*-forks (a *k*-fork can be defined by two paths of size *k* joined at one extremity by a common branching node) joining together in a pseudo-cycle the four k-mers *a*, *u*, *v*, and *b*. An efficient algorithm was implemented in the software TAKEABREAK to find such inversion breakpoints in the dBG while avoiding numerous false positives due to repeated sequences. The implementation has very limited memory impact and runtimes (Illumina reads simulated at 2×40x coverage from human chromosome 22 can be treated in less than 10 min, with less than 1 GB of memory).

#### MAPSEMBLER2

MAPSEMBLER2 [3] is a targeted assembly program. It takes as input one or more set(s) of NGS raw reads (FASTA or FASTQ, gzipped or not) and a set of input sequences, called the ‘starters’. All the input read sets are used together to assemble the neighbors of each of the starters provided. These neighbors are output either as simple sequences (a sequence is cut as soon as two choices occur during the assembly) or as a graph in which polymorphisms are shown.

**Fig. 5:**
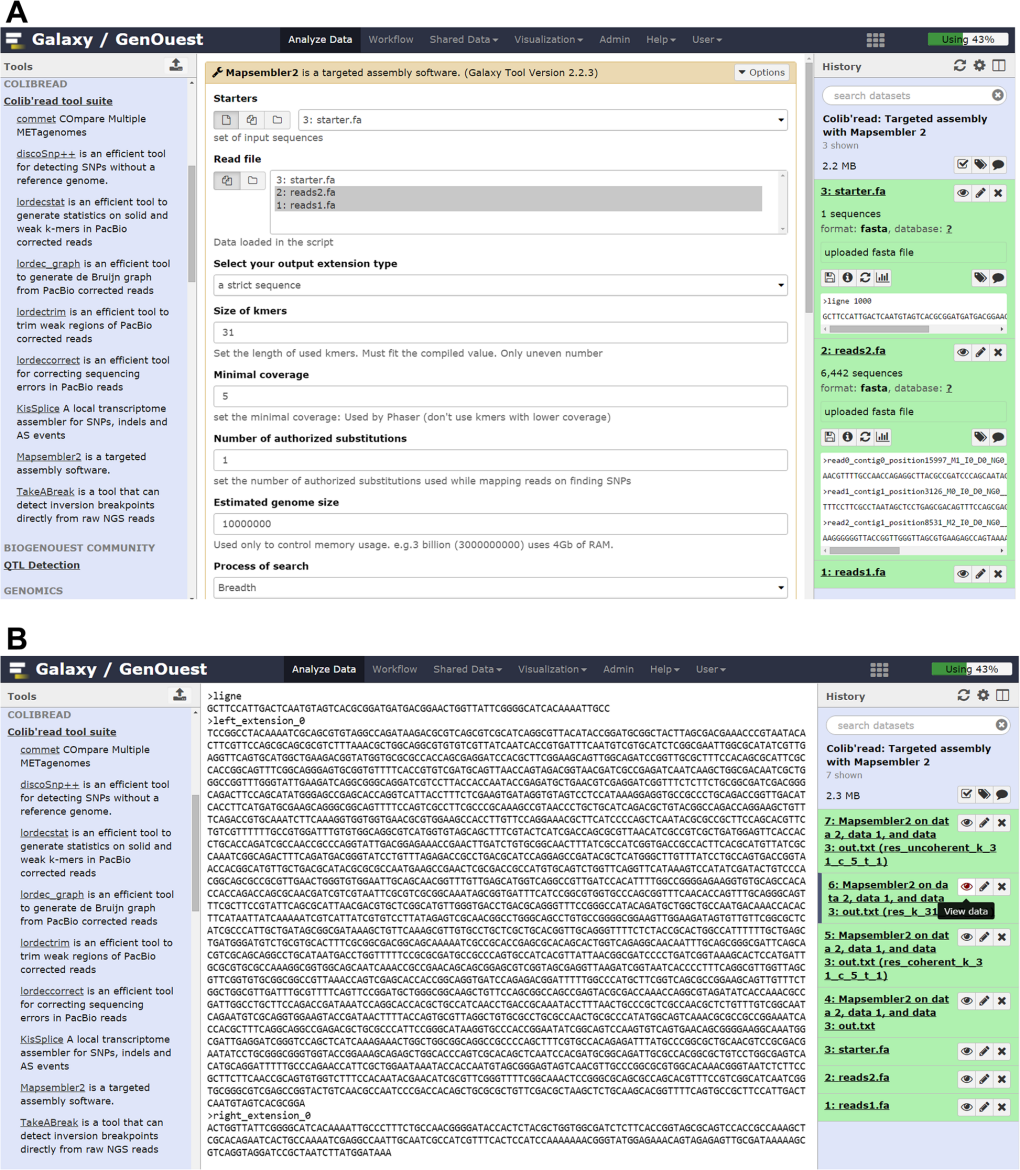
Running MAPSEMBLER2 on Galaxy. **a** MAPSEMBLER2 tool form allowing selection of input datasets and tool parameters. **b** MAPSEMBLER2 FASTA output

MAPSEMBLER2 may be used, for instance, for understanding the third-party assembly failures or chimeric assemblies, or for validating and visualizing the presence or absence of assumed polymorphism near one or several sequence(s) of interest (Fig. 5).

**Fig. 6:**
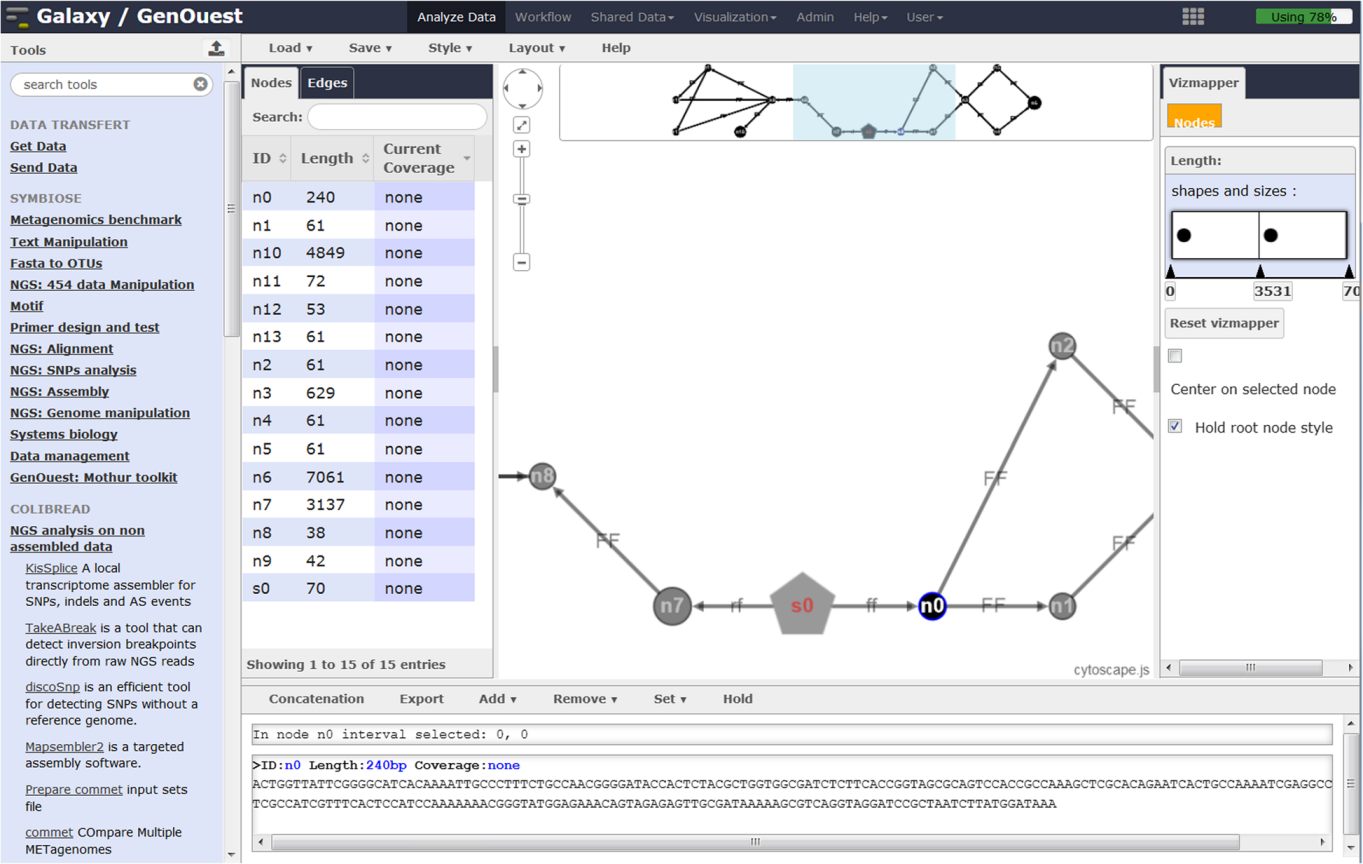
Running GSV on Galaxy. The JSON graph generated by MAPSEMBLER2 can be navigated, filter parameters used to modify the visualization aspect, and results exported

A special tool, called the ‘Graph of Sequence Viewer’ (GSV), was developed for visualizing and manipulating the graphs produced by MAPSEMBLER2, as shown in Fig. 6. Such graphs are in JSON format.

The visualization framework was designed to facilitate the interpretation of MAPSEMBLER2 outputs. Currently, this visualizer is compatible only with JSON format MAPSEMBLER2 outputs and with any dBG graph respecting the specific JSON characteristics. Further work will make GSV compatible with semantic web or systems biology tools to visualize, for example, RDF files or biological networks.

#### COMMET

COMMET [6] provides a global similarity overview between all datasets of a large metagenomic project. Directly from non-assembled reads, all-against-all comparisons are performed through an efficient indexing strategy. The results are stored as bit vectors, i.e. a compressed representation of read files, which can be used to further combine read subsets by common logical operations. Finally, COMMET computes a clusterization of the metagenomic datasets, which is visualized through dendrograms and heatmaps.

#### LORDEC

LORDEC [7] is a tool to correct sequencing errors in long reads obtained from third-generation high-throughput sequencing technologies [7]. Third-generation sequencing machines, especially PacBio, offer the advantage of delivering much longer reads than previous technologies (up to 20 Kb), often at the expense of sequence precision. Current estimates show that sequencing errors in PacBio reads average around 15 %, while traditional Illumina short reads exhibit an average error rate around 1 %. Moreover, PacBio sequencing suffers from a majority of insertion/deletion errors. It is thus necessary to correct these long reads before analysis, or at least during assembly, and different solutions have been proposed [19–22], but these approaches “require high computational resources and long running times on a supercomputer even for bacterial genome datasets”. [22].

In summary, LORDEC adopts a hybrid approach that takes advantage of the low error rate of short reads to correct the long ones. To avoid the computational bottleneck of all-against-all alignments, LORDEC builds the dBG of the short reads, and aligns long reads to the paths of the dBG. LORDEC exploits the fact that the dBG summarizes in a single structure the layout of short reads along the target DNA/RNA sequence. Hence, aligning a long read to the dBG allows the correction of erroneous sequence positions much more efficiently, and in a scalable manner. The dBG implementation of GATB allows LORDEC to process huge short reads libraries and to scale up to vertebrate or plant genome cases.

The LORDEC software offers several programs: the main one for correcting the long reads, and others for trimming and splitting corrected reads into corrected regions if needed. The output distinguishes these with lower vs upper cases. The value of *k* can be optimized by trying different values as low as possible around log4 (genome-length) (see [23] for an explanation).

Note that unlike the other tools, LORDEC addresses sequencing errors (which can be seen as technical artifacts) rather than biological events (such as variants, AS, etc.). Hence, LORDEC fulfills a need for preprocessing the read data before further analyses can extract biological information from it, as illustrated by the application to genome assembly described below.

### Applications

Table 2 presents several time and memory footprint results, showing how the tools presented scale up on large raw datasets.

#### *De novo* identification of alternative splicing events in human RNA-seq data with KISSPLICE

KISSPLICE was applied to a human dataset that consists of 32 million reads from human brain and 39 million reads from liver from the Illumina Body Map 2.0 Project (downloaded from the Sequence Read Archive, study accession number ERP000546, brain read accession numbers ERR030882 and ERR030890, liver read accession numbers ERR030887 and ERR030895). Even though KISSPLICE does not require a reference genome, we applied it to a case where an annotated reference genome is indeed available in order to be able to assess whether our predictions are correct.

KISSPLICE ran in three hours using less than 8 GB RAM and was able to identify 2,336 bubbles corresponding to AS events.

To assess whether these predictions were correct, we aligned both paths of each bubble to the human reference genome (version hg19) using STAR [24] with default parameters.

We found that for 132 bubbles (5.7 %), the two paths did not map to the same genomic location, suggesting that the bubbles were false positives. A manual inspection of these cases revealed that most of them were due to repeats.

Among the bubbles where both paths mapped to the same location, we found that 1,714 (81 %) corresponded to annotated AS events, according to Ensembl v75 annotation [25]. In contrast, 398 (19 %) corresponded to putative novel AS events, with at least one splice site not annotated before. Out of those 398 cases, 78 % (vs 97 % of them for the annotated splice sites) were canonical (GT-AG), and 22 % were non-canonical. An issue common to all transcriptome assemblers is that genomic indels, when located in transcribed regions, can be confused with AS events since they also generate bubbles in the dBG. In the presence of a reference genome, we can tell them apart easily as one path will map in two blocks and the other in one block. Using this criterion, we found that half of the non-canonical novel AS events were indeed indels (49 %). The remaining 51 % were composed of GC-AG, novel splice sites, and some mapping or assembly errors.

To summarize, we find that out of 2,336 bubbles reported by KISSPLICE, 76 % are AS events annotated in Ensembl, 14 % are AS events, not annotated but canonical, 2 % are AS events, not annotated and not canonical, 2 % are genomic indels, and 5 % are repeat-associated false positives.

Furthermore, we reported in [2] that KISSPLICE is more sensitive than Trinity, a widely used full-length transcriptome assembler, for calling AS events. The recent developments [18] in KISSPLICE show a particular sensitivity enhancement compared to Trinity in the case where there is still some pre-mRNA in the sample to be analyzed. The presence of pre-mRNA can in practice vary from 5 – 15 % depending on the protocol to isolate mRNA (total RNA vs nuclear, polyA+ vs polyA-). This can have a large impact on the assembly since introns are usually repeat-rich and the repeats are currently poorly handled by transcriptome assemblers.

#### Assessing DISCOSNP recall on real read sets from *Saccharomyces cerevisiae*

DISCOSNP was applied to a set of biologically validated SNPs predicted from an artificial evolution study on *S. cerevisiae* [26]. Twenty-four read sets (corresponding to three populations) were downloaded from the NCBI Sequence Read Archive (with the accession number SRA054922) and processed to remove barcode and adapter sequences as in the initial study. DISCOSNP was run independently on the three populations studied. For each population, DISCOSNP was applied to the eight read sets corresponding to eight time points, with the default parameters and *c*=11. In this framework, DISCOSNP recall could be evaluated on real read datasets. As shown Table 3, among the 29 reference-validated isolated SNPs, 27 were predicted by DISCOSNP, thus giving an estimated recall of 93.1 %. Overall, this experiment demonstrates the good performance of DISCOSNP at discovering SNPs from pooled samples, even those with low allelic frequencies: most of the reference SNPs were reported in the initial study with a minor allele frequency (MAF) lower than 20 %. Note that no SNP with a MAF lower than 10 % was experimentally validated, so we could not assess the recall on these very low frequency SNPs.

**Table 3:** Isolated SNPs found in *S. cerevisiae* and validated in [26]

First population studied				
(5 found among 6)				
Chromosome	Position	Ref	Alt	Predicted by DISCOSNP
1	39425	A	G	Yes
3	235882	C	A	Yes
4	1014740	G	C	Yes
6	71386	G	C	Yes
12	200286	C	T	Yes
15	438512	A	C	No
Second population studied				
(9 found among 9)				
Chromosome	Position	Ref	Alt	Predicted by DISCOSNP
1	39261	G	A	Yes
4	1014763	T	G	Yes
4	1014850	T	A	Yes
6	71813	A	C	Yes
7	146779	T	C	Yes
10	179074	C	A	Yes
12	162304	A	T	Yes
14	681026	T	G	Yes
15	412148	G	T	Yes
Third population studied				
(13 found among 14)				
Chromosome	Position	Ref	Alt	Predicted by DISCOSNP
1	191184	A	G	No
2	521881	C	T	Yes
4	1014981	A	T	Yes
4	1015077	G	T	Yes
6	70913	C	T	Yes
9	401526	G	A	Yes
10	250988	G	A	Yes
10	619870	G	T	Yes
11	64697	T	C	Yes
11	434707	A	G	Yes
12	404866	G	T	Yes
15	174575	T	G	Yes
15	1013813	C	A	Yes
16	79761	T	G	Yes

chr16:581589 mutation in experiment E2, originally presented in [26], is not reported in this table, as it could not be validated

#### Targeted assembly of *S. cerevisiae* using MAPSEMBLER2

**Fig. 7:**
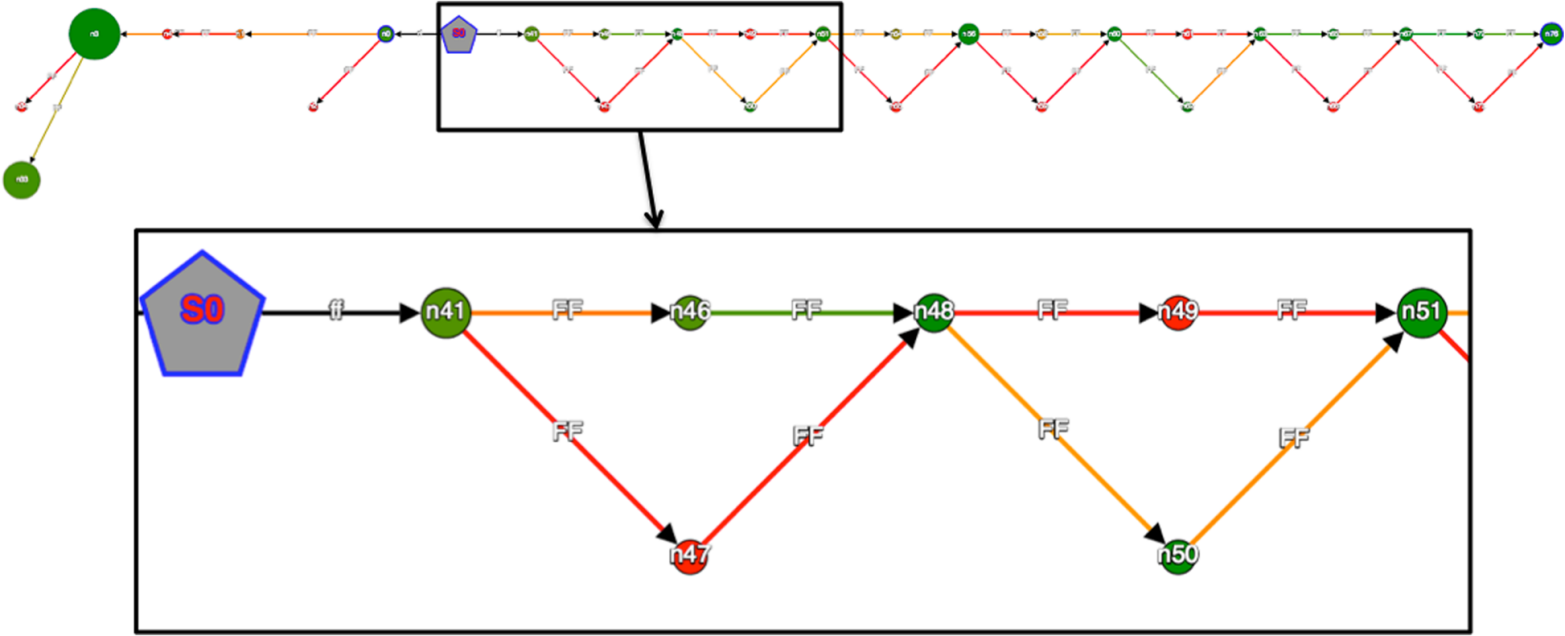
MAPSEMBLER2 output graph obtained from the *Saccharomyces cerevisiae* dataset visualized using GSV. A zoom is proposed for visualizing first nodes. The grey node is the starter. Node size depicts the length of the sequence stored by the node. The node and edge colors depict the read coverage (here for one among all datasets) of the sequence stored by the node. The ‘bubbles’ seen on the right of the starter witness the presence of SNPs and small indels in the datasets. Note that by changing the choice of the read set selected for visualizing the coverage (node and edge colors), one can deduce the heterozygous or homozygous nature of these variants

MAPSEMBLER2 was applied to the *S. cerevisiae* dataset previously described in the DISCOSNP section. Biological validation of several identified SNPs is presented in a recent study [26]. As a starter, we selected a sequence fragment of length 63 bp, occurring at position 1,014,600 on chromosome 4. This starter, GGGG TTTTTCAACTGAATGTTCTTCAATAAAGCCTTTTT CAGAAGCGATTTTGTTTCTGTGCT, occurs near a set of SNPs validated in the [26] study. The graph produced (Fig. 7) enables the retrieval of these validated SNPs, and also allows a check of the coverages of their two alleles in each of the 16 input read sets. Additionally, this graph also enables the detection of three SNPs and an indel that were not detected in the mapping pipeline used in [26].

#### Metagenomics global similarity overview of five gut metagenomes with COMMET

The MetaSoil study focuses on untreated soils of the Park Grass Experiment, Rothamsted Research, Hertfordshire, UK. One of the goals of this study is to assess the influence of depth, seasons, and extraction procedure on the sequencing [27]. To achieve this, the 13 metagenomes from MetaSoil, two additional soil metagenomes, and a seawater metagenome were compared at the functional level using MG-RAST [28]. This approach identified 835 functional subsystems present in at least one of the metagenomes that were used for clustering samples.

This study was reproduced with COMMET on all available metagenomes. The generated bit vectors total 68 MB, while the explicit representation of the FASTA results requires 6.4 GB. The storage footprint is thus divided by a factor of 100. This ratio is even higher if using FASTQ format or if dealing with larger read files. The COMMET computation time was 828 min.

**Fig. 8:**
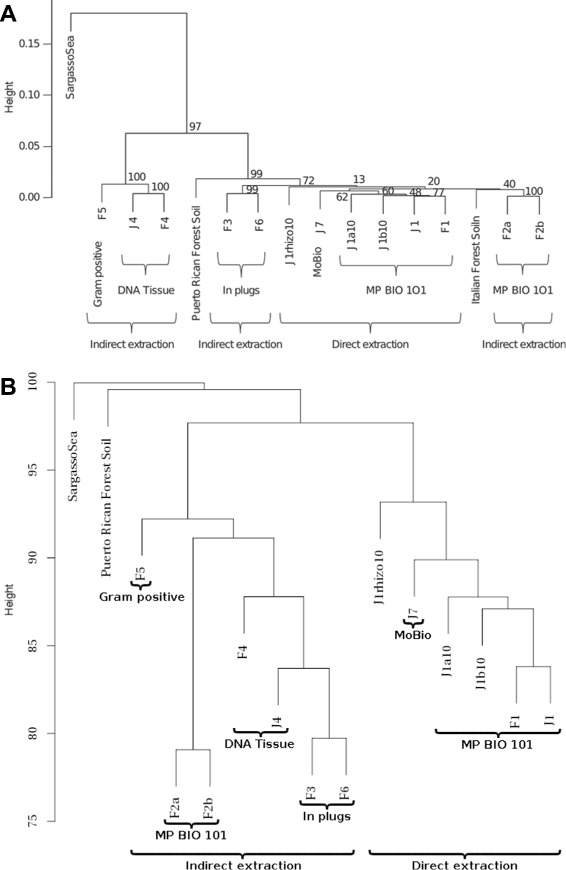
Dendrograms from MetaSoil study. **a** Fig. from [29] showing the cluster tree, constructed using Euclidean distances, confronting 13 samples others soil metagenomes (Puerto Rican Forest soil and Italian Forest Soil) and a metagenome from Sargasso Sea (SargassoSea). DNA extraction methods are indicated. Thus, “MP BIO 101” means Fast prep MP Bio101 Biomedical, Eschwege, Germany, “In plugs” means indirect lysis in plug, “DNA Tissue” means Nucleospin Tissue kit, “MoBio” means MoBio Powersoil DNA Isolation Kit (Carlsbad, CA, USA) and finally “Gram positive” for the Gram-positive kit **b** COMMET analyses, comparing the same 15 samples

Although COMMET uses another metric, the dendrogram produced is highly similar to the MetaSoil one (see Fig. 8), and enables us to reach the conclusion that two metagenomic samples processed with the same extraction procedure share more similarities at the functional level than two samples processed with different extraction procedures [29].

#### LORDEC: impact of read correction on genome assembly

To summarize, experiments on real data taken from bacterial and yeast species, up to the case of a vertebrate genome, show that LORDEC achieves a quality at least as high as that of available state-of-the-art methods, while usually being an order of magnitude faster. Exploiting the GATB implementation of the dBG makes it by far the most scalable and economical option in terms of memory usage: LORDEC can process large datasets on a standard computer.

To assess the impact on the assembly quality of the PacBio reads correction performed with LoRDEC, we compared the assemblies obtained from corrected reads to those computed from uncorrected reads. For this purpose, we used public datasets from the *Escherichia coli* and *S. cerevisiae* genomes (see Table 4). For each genome, we corrected with LoRDEC the PacBio reads using a set of short reads (with parameters *k*=19 and *s*=3, and default values for the other parameters). We then separately assembled corrected and uncorrected PacBio reads using the ABySS assembler with different values of *k* [30] (see the details in Additional file 1). The results are given in Table 5.

**Table 4:** Datasets used to evaluate the efficiency and impact of LoRDEC read correction on the assembly

	*E. coli*	Yeast	
Reference organism			
Name	*Escherichia coli*	*Saccharomyces cerevisiae*	
Strain	K-12 substr. MG1655	W303	
Reference sequence	NC_000913	S288C	
Genome size	4.6 Mbp	12 Mbp	
PacBio Data			
Accession number	PacBio reads	DevNet PacBio	
Number of reads	75152	261964	
Average read length	2415	5891	
Max. read length	19416	30164	
Number of bases	181 Mbp	1.5 Gbp	
Coverage	30 ×	129 ×	
Illumina Data			
Accession number	Illumina reads	SRR567755	
Number of reads (millions)	11	2.25	
Read length	114	100	
Number of bases	1.276 Gbp	225 Mbp	
Coverage	277 ×	18 ×	

For the short read data of yeast, we used only half of the available reads. The reference yeast genome is available from [40]

**Table 5:** Comparison of the assemblies obtained for *E. coli* and *S. cerevisiae* from either uncorrected or corrected PacBio reads

	*E. coli* (*k*=64)		*S. cerevisiae* (*k*=51)	
Statistical metrics	Corrected	Uncorrected	Corrected	Uncorrected
Number of contigs	2349	1721	61496	39127
Number of contigs ≥ 1 kbp	321	0	1657	0
Genome coverage (%)	98	0	91	0
Total length (Mbp)	4.71	0.12	15.00	2.39
Largest contig (bp)	93000	127	52444	378
GC (%)	50.19	3.77	38.75	40.00
N50	23473	69	6943	57

The genome coverage accounts only for contigs longer than 1kbp. With uncorrected reads, the N50 remains close to the *k*-mer length (whatever the value of *k*); this strongly suggests that ABySS fails to assemble uncorrected reads. On the contrary, the metrics with corrected PacBio reads indicate that it yields satisfactory assemblies for both genomes

With uncorrected reads, and whatever the value of *k*, ABySS (v1.3.2) yields an assembly whose N50 value is close to *k* on both genomes, and with the longest contig below 400 bp. With PacBio reads corrected using LoRDEC, ABySS assemblies cover respectively 98 and 91 % of *E. coli* and *S. cerevisiae* genomes with respectively 321 and 1,657 contigs larger than 1 kbp (*k*=64 and *k*=51). Their N50 values reach 23 and 6.9 kbp respectively, while the largest contigs are 93 and 52 kbp long. Moreover, when aligning (using BLASTN, NCBI-BLAST-2.2.29+, with a reward of 1 and a penalty of −3) the contigs longer than 1 kbp against the reference genome, only 2.6 % of yeast contigs lack similarity, while all contigs of *E. coli* could be aligned.

The genome coverage and N50 values show that ABySS did not succeed in assembling any of the uncorrected PacBio datasets, while it yields satisfactory assemblies (i.e. sets of contigs) with the same PacBio reads that were corrected with LoRDEC [7]. Without correction, an assembly obtained from a traditional program is useless; clearly, LoRDEC correction has a strong impact on the assembly quality of PacBio reads. Moreover, the correction is faster than the assembly and simplifies the latter. Importantly, the results suggest that hybrid correction using LoRDEC makes PacBio reads amenable to a classical de Bruijn graph assembly approach. LoRDEC has also been applied to MinION reads obtained with Oxford Nanopore technology and we observed a strong improvement in the mapability of the reads once corrected. More precisely, mapping the reads of an *E. coli* dataset on the reference genome with NucMer/Quast [31], we found that, while none of the raw MinION reads could be fully aligned, 2383 out of 2749 corrected reads were fully aligned on the genome, thereby covering 96 % of the genome. In addition, corrected reads could also be assembled with a de Bruijn-based approach.

### Galaxy integration

Tools integration was made following Galaxy [8–11] team recommendations on tool configuration syntax [32] as well as on tool shed administration and use [33]. For each Galaxy tool repository, two packages are defined, one for dependencies, the other for descriptor and wrapper if required. We used the GenOuest Galaxy development tool shed and development Galaxy instance to create and test the tools. GSV was originally a standalone web tool for MAPSEMBLER2 output graph visualization. Adding this tool as a visualization tool on Galaxy [34] was done following Galaxy [8–11] team instructions. Briefly, an XML configuration file is first created for the visualizer to define a link between a dataset and GSV. This GSV.xml file calls a Python GSV.mako template allowing dynamic generation of HTML and javascript codes. Finally, a GSV.js script is called to manage Galaxy file dependencies, objects, and visualization library.

### Tool suite sharing

GUGGO Tool Shed [12, 13] was used to disseminate Colib’read Galaxy repositories. Corresponding tools are installed on our production Galaxy instance [35, 36], allowing scientists to use Colib’read tools freely after registration on the GenOuest core facility [37]. As we join a dependencies package to our tools, Galaxy instance administrators can easily install either Galaxy tools (i.e. description files and wrappers) or Colib’read binaries and dependencies without any command line typing.

## Conclusion

We propose bioinformatics tools dedicated to raw NGS data analyses for DNA-seq, RNA-seq and metagenomics studies. Thanks to the Galaxy platform, we easily made this tools suite available to life scientists, regardless their level of programming skills. Colib’read tools thus inherit reproducibility and accessibility support from Galaxy. Moreover, with the growing number of bioinformatics core facilities hosting Galaxy servers, tool shed usage enhances tools descriptors, binaries and dependencies sharing. This tools suite allows life scientists to find candidates that cannot be found with classical assembly-based approaches. Moreover, the algorithm developments described in this paper enhance the optimization and the management of the use of computing resources, in a time where these resources can not match the pace imposed by the NGS data deluge. Applications presented in this paper illustrate the low memory footprint of the six tools developed within the Colib’read framework, as well as their scalability. In replacement or combination with classical approaches, we thus propose to deal with higher amounts of information by using efficient computation strategies for NGS data.

## Availability and requirements

**Project name:** Colib’read project **Project home page:** [38] **Operating system(s):** Platform independent **Programming language:** C++ **Other requirements:** GATB core **License:** A-GPL and CeCILL **Any restrictions to use by non-academics:** None

## Availability of supporting data

All data sets supporting the analyses are available from the GigaScience GigaDB repository [39].

## Additional file

Additional file 1
**Evaluation of the efficiency and the impact of the LoRDEC correction on** ***E. coli*** **and** ***S. cerevisiae*** **genome public data.** (PDF 131 kb)
