# Supplementary material for: Colib’read on galaxy: a tools suite dedicated to biological information extraction from raw NGS reads
Source: Gigascience. 2016 Feb 11;5:9. doi: 10.1186/s13742-015-0105-2 (PMC4750246; doi:10.1186/s13742-015-0105-2)
Supplement: Additional file 1 — Evaluation of the efficiency and the impact of the LoRDEC correction on E. coli and S. cerevisiae genome public data. (PDF 131 kb) [file 13742_2015_105_MOESM1_ESM.pdf]

LoRDEC evaluation: supplementary File - Data and Parameters

Y. Le Bras et al.

To evaluate the efficiency and the impact of the LoRDEC correction at the assembly level, we used public data of two genomes *E. coli* and *S. cerevisiae* (see Table 1). Pacbio reads correction was carried out using LoRDEC (v 0.3) tool using $-$`k19` and $-$`s3` and other parameters by default. Then, we ran the ABySS assembler (v 1.3.2) to assemble Pacbio reads using two k-mer sizes for each genome [1]. Blastn (ncbi-blast-2.2.29+) was used to align the obtained contigs against their reference genome with reward of 1 and a penalty of -3.

We observed in the absence of PacBio reads correction, the distribution of kmer abundance never exceed 65 while after correction, the same data showed a maximal abundunace value of 250 (see Figure 1). This result attests the efficiently of LoRDEC to correct PacBio reads.

References

[1] Jared T. Simpson, Kim Wong, Shaun D. Jackman, Jacqueline E. Schein, Steven J.M. Jones, and Inanç Birol. Abyss: A parallel assembler for short read sequence data. *Genome Research*, 19(6):1117–1123, 2009.

	E. coli	Yeast
**Reference organism**		
Name	*Escherichia coli*	*Saccharomyces cerevisiae*
Strain	K-12 substr. MG1655	W303
Reference sequence	NC_000913	S288C
Genome size	4.6 Mbp	12 Mbp
**PacBio Data**		
Accession number	PacBio reads	DevNet PacBio
Number of reads	75152	261964
Avg read length	2415	5891
Max read length	19416	30164
Number of bases	181 Mbp	1.5 Gbp
Coverage	30x	129x
**Illumina Data**		
Accession number	Illumina reads	SRR567755
Number of reads (millions)	11	2.25
Read length	114	100
Number of bases	1.276 Gbp	225 Mbp
Coverage	277x	18x

Table 1: Datasets used to evaluate the efficency and impact of LoRDEC read correction on the assembly. For the short read data of yeast, we used only half of the available reads. The reference yeast genome is available at this site.

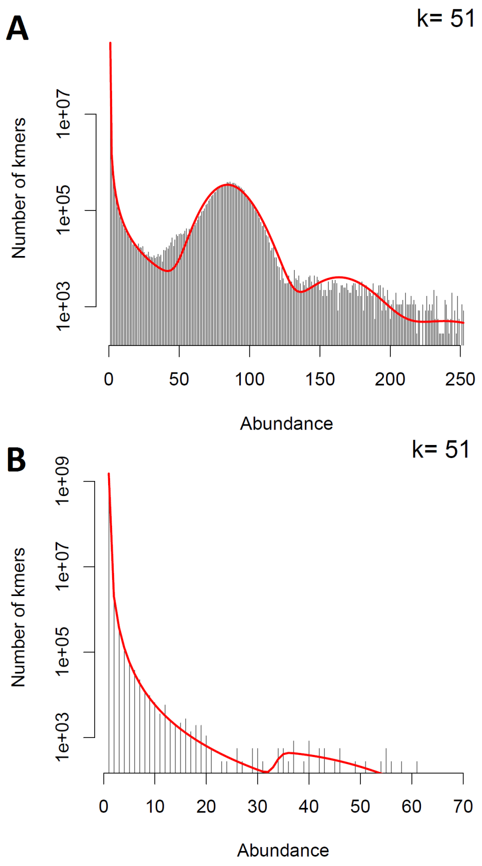

Figure 1: Distribution of the number of distinct *k*-mers (y-axis) with respect to their abundance (x-axis) in yeast PacBio reads with (A) and without (B) error correction. Without correction very few *k*-mers exceeds an abundance of 20, while after correction there is a peak representing many *k*-mers with abundance around 75. The graphics were obtained with K-mer genie.
